# Stereotactic body radiation therapy (SBRT) following Yttrium-90 (^90^Y) selective internal radiation therapy (SIRT): a feasibility planning study using ^90^Y delivered dose

**DOI:** 10.1088/1361-6560/acbbb5

**Published:** 2023-03-10

**Authors:** Stephen F Mee, Daniel F Polan, Yuni K Dewaraja, Kyle C Cuneo, Joseph J Gemmete, Joseph R Evans, Theodore S Lawrence, Janell S Dow, Justin K Mikell

**Affiliations:** 1 Department of Radiology, University of Michigan, Ann Arbor, MI, United States of America; 2 School of Medicine, Wayne State University, Detroit, MI, United States of America; 3 Department of Radiation Oncology, University of Michigan, Ann Arbor, MI, United States of America

**Keywords:** hepatocellular carcinoma, Yttrium-90, radioembolization, SBRT, SIRT, Y-90, radiation therapy

## Abstract

*Objective*. ^90^Y selective internal radiation therapy (SIRT) treatment of hepatocellular carcinoma (HCC) can potentially underdose lesions, as identified on post-therapy PET/CT imaging. This study introduces a methodology and explores the feasibility for selectively treating SIRT-underdosed HCC lesions, or lesion subvolumes, with stereotactic body radiation therapy (SBRT) following post-SIRT dosimetry. *Approach*. We retrospectively analyzed post-treatment PET/CT images of 20 HCC patients after ^90^Y SIRT. Predicted tumor response from SIRT was quantified based on personalized post-therapy dosimetry and corresponding response models. Predicted non-responding tumor regions were then targeted with a hypothetical SBRT boost plan using a framework for selecting eligible tumors and tumor subregions. SBRT boost plans were compared to SBRT plans targeting all tumors irrespective of SIRT dose with the same prescription and organ-at-risk (OAR) objectives. The potential benefit of SIRT followed by a SBRT was evaluated based on OAR dose and predicted toxicity compared to the independent SBRT treatment. *Main results*. Following SIRT, 14/20 patients had at least one predicted non-responding tumor considered eligible for a SBRT boost. When comparing SBRT plans, 10/14 (71%) SBRT_boost_ and 12/20 (60%) SBRT_alone_ plans were within OAR dose constraints. For three patients, SBRT_boost_ plans were within OAR constraints while SBRT_alone_ plans were not. Across the 14 eligible patients, SBRT_boost_ plans had significantly less dose to the healthy liver (decrease in mean dose was on average ± standard deviation, 2.09 Gy ± 1.99 Gy, ) and reduced the overall targeted PTV volume (39% ± 21%) compared with SBRT_alone_. *Significance*. A clinical methodology for treating HCC using a synergized SIRT and SBRT approach is presented, demonstrating that it could reduce normal tissue toxicity risk in a majority of our retrospectively evaluated cases. Selectively targeting SIRT underdosed HCC lesions, or lesion subvolumes, with SBRT could improve tumor control and patient outcomes post-SIRT and allow SIRT to function as a target debulking tool for cases when SBRT is not independently feasible.

## 1. Introduction

Liver cancer remains a leading contributor to cancer mortality globally, and the incidence rate has substantially increased over the last two decades (Sung *et al* 2021). Selective internal radiation therapy (SIRT) with ^90^Y microspheres has been demonstrated as a safe and effective therapy for inoperable primary liver malignancies (predominately HCC) and liver metastases, with a post-treatment median overall survival (OS) time of 5.6–16.4 months (Saini *et al* 2019). However, despite initially promising clinical results, recent phase III trials comparing ^90^Y SIRT with a standard therapy chemotherapeutic agent have failed to demonstrate any improvement in patient outcomes (Vilgrain *et al* 2017, Ricke *et al* 2019).

Dosimetry for SIRT planning can be performed pre-therapy by utilizing SPECT/CT imaging of ^99m^Tc macroaggregated albumin (MAA) particles, which serves as a surrogate for ^90^Y microspheres (Garin *et al* 2012). Recent studies have demonstrated that patient outcomes are correlated with mean tumor dose (TD) and that outcomes can be stratified based on a threshold TD for achieving a response (Giammarile *et al* 2011). The recent DOSISPHERE-01 study demonstrated a benefit to overall survival when ^99m^Tc MAA-based planning is used to target a specific TD (Garin *et al* 2021). The authors reported a 16 month survival advantage for HCC patients treated with a plan to deliver >205 Gy to a large (>7 cm) index lesion compared with standard treatment that is only planned based on dose to the liver volume (tumoral and nontumoral liver) expected to be perfused by microspheres . However, pre-therapy surrogate dosimetry relies on the assumption that it accurately represents the final ^90^Y microsphere distribution as delivered clinically, leading to varied results (Mikell *et al* 2019). For more accurate dosimetry, the microsphere distribution can be analyzed after treatment with emission-based imaging ( ^90^Y PET or Bremsstrahlung SPECT) and potentially soon through CT imaging with the development of radiopaque microspheres (Kappadath *et al* 2018, Dewaraja *et al* 2020, Henry *et al* 2022). Current clinically available dosimetry performed with PET imaging takes advantage of the superior spatial resolution and absolute quantification capabilities compared to SPECT, which is especially relevant for lesion dosimetry.

Another emerging treatment modality for HCC has been stereotactic body radiation therapy (SBRT), with a recent review demonstrating excellent local control with low liver toxicity (Gerum *et al* 2019). One phase 2 trial demonstrated a 2-year local control and OS of 97% and 84%, respectively, amongst 65 HCC patients treated with SBRT (Jang *et al* 2020). However, for cases with a large tumor burden, liver toxicity can become a major limitation for SBRT when attempting to balance lesion control with dose to the surrounding normal liver tissue (NLT) (Kim and Jung 2017).

Given the demonstrated importance of dose thresholds for lesion response in SIRT and effectiveness of SBRT, one way to improve patient outcomes could involve supplementing (boosting) lesions underdosed by SIRT with SBRT. Similar types of combined therapies, such as using external beam radiotherapy (EBRT) with brachytherapy, are an established and effective treatment regimen for other cancer sites (Hsu *et al* 2021). A combination SBRT/SIRT therapy could be desirable for the liver, as cases with high tumor burden could first be debulked by SIRT, then followed up with SBRT where SBRT alone may have not been initially possible without exceeding NLT constraints. This approach makes use of the fact that SIRT is inherently sparing of healthy tissue due to preferential uptake of ^90^Y in lesions relative to healthy liver and the limited range of the beta particles. There are prior studies reporting the combination of ^90^Y SIRT and EBRT delivered to the liver in the same patients, with no major toxicities noted (Abbott *et al* 2020, Liu *et al* 2021). However, to our knowledge this is the first study to propose direct usage of delivered ^90^Y dose maps, with predicted tumor responses, to create EBRT plans that dynamically conform to SIRT underdosed tumor regions.

The purpose of our study was to determine the feasibility of delivering a supplemental boost with SBRT to HCC tumors or regions of tumors that did not receive sufficient SIRT dose predictive of a therapeutic response. Specifically, utilizing ^90^Y PET/CT dose maps following SIRT and previously published tumor control probability (TCP) curves, we use a dose-painting approach to generate hypothetical SBRT_boost_ plans that target only lesion volumes that were underdosed by SIRT. These boosts are compared with SBRT_alone_ plans that target all lesions assuming that no SIRT was delivered. We expect that delivering tumoricidal SBRT doses to tumor regions underdosed by SIRT could improve patient outcomes by controlling disease or reducing tumor burden to enable further directed therapy.

## 2. Methods and materials

### 2.1. Patient data and ^90^Y dosimetry

We performed a retrospective review of patients treated on a prior University of Michigan institutional review board approved research study, from December 2014 to February 2020, in which patients with HCC or liver metastases, after signing informed consent, underwent post-therapy PET/CT imaging for direct ^90^Y dosimetry following SIRT. All patients received standard-of-care SIRT consistent with the manufacturer’s recommendations (Therasphere™, Boston Scientific, Marlborough, MA). For the current study, patient selection was limited to patients with HCC and at least one lesion >2 cm^3^, a threshold chosen based on the limited spatial resolution of ^90^Y PET. Of the 77 patients who participated in the previous study, 20 met the selection criteria for this study.

Detailed information regarding ^90^Y PET/CT imaging following the SIRT procedure and dosimetry calculations are available in a prior study and are briefly summarized here (Dewaraja *et al* 2020). ^90^Y PET/CT time-of-flight imaging was performed on a Siemens Biograph^TM^ mCT (Siemens Healthineers, Erlangen, Germany) with acquisition times of approximately 30 min and continuous bed motion covering the entire liver. The non-contrast CT was acquired in a free-breathing state in the low-dose mode. ^90^Y physical absorbed dose maps were calculated from the ^90^Y PET/CT using an in-house Monte Carlo algorithm and accounting for physical decay (Wilderman and Dewaraja 2007). Isodose contours were created and saved from the dose map for use in SBRT planning, as described in a later section.

For each case, the largest HCC lesions (up to 5 total and >2 cm^3^) were manually segmented by a radiologist specializing in hepatic malignancies. If lesions were well visualized anatomically on the post-SIRT PET/CT, segmentation was performed directly to avoid the requirement for image registration. For lesions that were poorly visualized, segmentation was performed on pre-SIRT diagnostic contrast-enhanced CT or MR imaging. These lesion contours were then transferred to the ^90^Y dose map using a rigid registration between the pre-SIRT diagnostic imaging and post-SIRT PET/CT. Contour locations were fine-tuned by the radiologist when mis-registration was evident. Mean TDs were calculated with sphere phantom-based recovery coefficients applied for partial volume correction (Dewaraja *et al* 2020).

### 2.2. SBRT boost planning (SBRT_boost_) based on SIRT TCP

**Figure 1.**
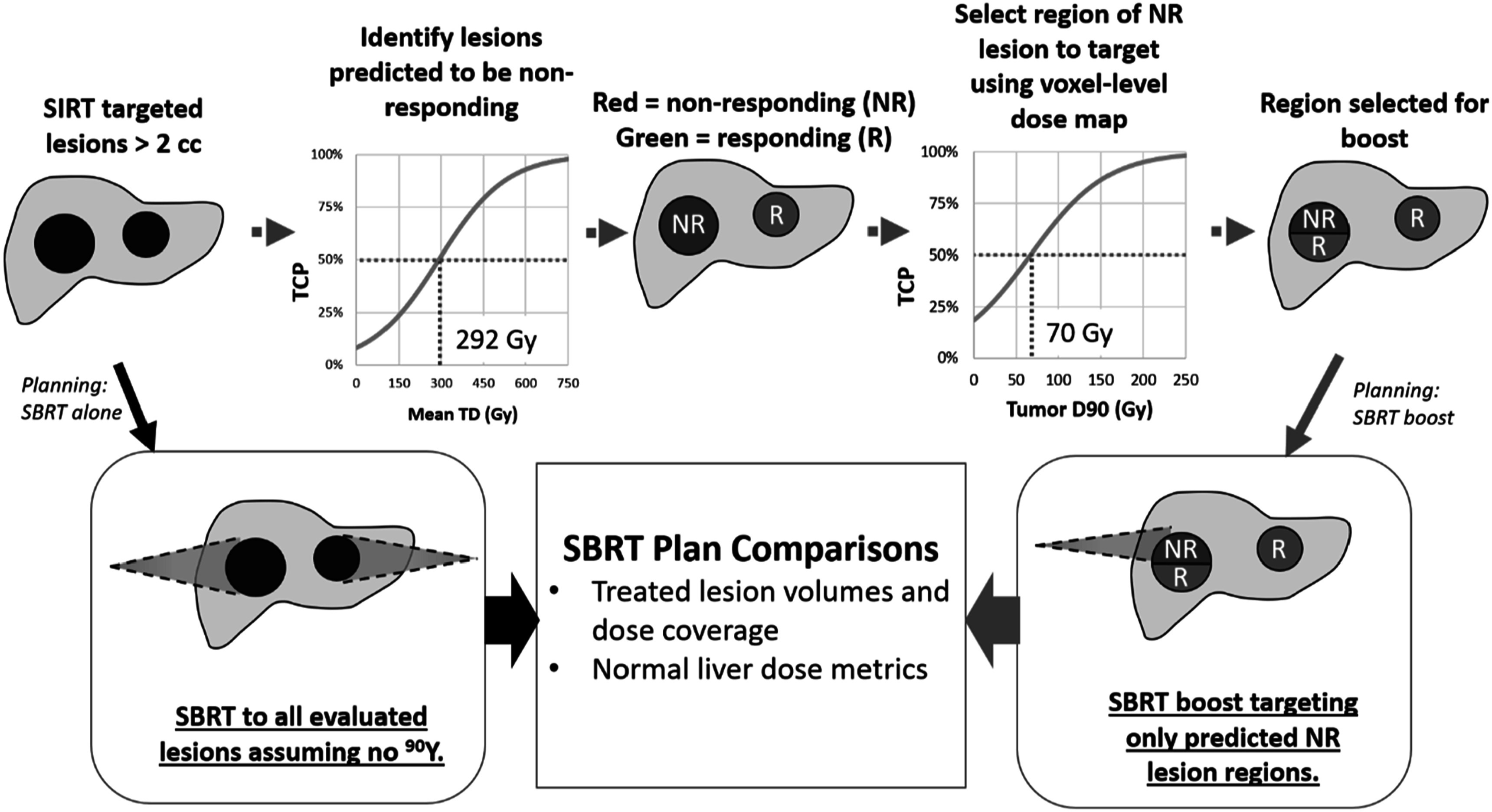
The target volume selection process for SBRT_boost_ (blue arrows) and SBRT_alone_ (black arrows). SBRT_boost_ plans are designed to target only lesions with mean SIRT dose of <292 and then subregions of those lesions that received <70 Gy from SIRT.

Targets for SBRT boost planning were determined using two SIRT TCP models based on individualized dosimetry. We first used a previously published TCP logit model of lesion response versus ^90^Y PET-derived TD, shown in the upper branch of figure 1 (Dewaraja *et al* 2020). In this study, response to SIRT was defined as a complete or partial response to modified RECIST criterion at 3–6 months post-SIRT (Dewaraja *et al* 2020). In the current study we chose a TCP of 50%, which corresponds to 292 Gy, as the threshold criteria for selecting lesions to be targeted by the SBRT boost. Therefore, lesions that received at least a mean SIRT TD of 292 Gy were excluded from the SBRT boost while lesions below this threshold were eligible.

Based on our SIRT dose maps, and as reported in other studies analyzing SIRT personalized dosimetry, dose heterogeneity across underdosed lesions in SIRT can be considerably large (Willowson *et al* 2017, Dewaraja *et al* 2020). Therefore, once lesions that could benefit from SBRT boosting are identified, it may be advantageous to specifically target the tumor subregions that were underdosed with SIRT using a dose painting approach. To facilitate this technique, we generated a new TCP logit-model, also shown in figure 1, based on modified RECIST response versus the coverage metric D90 (i.e. the minimum dose to 90% of the tumor volume). This model was created through logistic regression independently of the mean SIRT dose TCP model and is based on D90 values that were directly calculated from the SIRT absorbed dose map without recovery coefficient applied (as that correction is only suitable for mean dose). Similar to the mean SIRT dose TCP model, we selected 50% as the basis for generating a dose threshold for SBRT boost targeting. The SIRT D90 value yielding 50% TCP was 70 Gy. Therefore, for all boost-eligible lesions, subregions were targeted based on an added 70 Gy threshold from SIRT. In summary, targets for SBRT planning were determined by first excluding lesions based on a mean ^90^Y SIRT TD threshold of 292 Gy, then further excluding regions of eligible lesions that received at least 70 Gy from SIRT. An overview of this target selection process (also referred to SBRT target debulking) is shown in figure 1.

The clinical ^90^Y TD maps, lesion contours, and corresponding CT from the PET/CT were transferred to Eclipse^TM^ v15.6 (Varian Medical System, Palo Alto, CA) for SBRT treatment planning. Inverse planning was performed directly on the CT of the PET/CT (using the Varian default CT electron density calibration), as no simulation CTs were available for this retrospective planning study. The gross tumor volumes (GTV) of all SBRT boost-eligible lesions minus the SIRT 70 Gy isodose contour were used to create new SBRT targets for the boost. Although the CTs were acquired in a free-breathing state, motion management was not considered in the absence of a typical pre-SBRT motion assessment from a 4D-CT. Therefore, SBRT planning was performed without use of an internal target volume (ITV). Planning target volumes (PTV) were created directly expanding the boost-eligible target regions by 5 mm left/right/posterior/anterior and 8 mm superior/inferior. To enable SBRT planning, relevant organs-at-risk (OAR) contours for each patient were defined on the CT of the PET/CT. Liver contours were generated semi-automatically using a commercially available deep-learning algorithm (ProtegeAI^TM^, MIM Software Inc., Beachwood, OH) followed by manual edits where necessary. Normal liver volume was defined as the liver minus all segmented lesions, regardless of eligibility for SBRT boost. Other OARs were delineated manually, and all planning structures were reviewed by an experienced medical physicist prior to treatment planning.

Photon SBRT planning consisted of 2–4, 6 MV flattening filter free coplanar arcs using beam data from a Varian TrueBeam with a high definition multileaf collimator, and were optimized and calculated using standard clinical algorithms (Photon Optimizer v13.6.23 and Anisotropic Analytical Algorithm v13.6.23, Varian Medical System, Palo Alto, CA). Based on our clinical practice for HCC patients, the prescription dose for each PTV was uniformly set to 36 Gy in 3 fractions regardless of prior SIRT dose. Similarly, standard clinical SBRT OAR constraints used by our institution were included as objectives in the optimization process for these single volumetric modulated arc (VMAT) plans. These included NLT objectives that prioritized mean dose < 9 Gy, and the sparing of a hepatic reserve (CV_15Gy_ > 700 cm^3^, the volume of NLT that receives less than 15 Gy), both of which are derived from QUANTEC limits for radiation-induced liver disease (RILD) (Pan *et al* 2010). To maintain consistency for optimization and preserve similar PTV coverage between plans, V_38 Gy_ ≥ 99.9 was used as a standard optimization objective that functioned to continuously push the optimizer to maintain coverage in the presence of competing OAR objectives.

### 2.3. SBRT only planning (SBRT_alone_)

For comparison purposes, another SBRT plan was generated ignoring personalized dosimetry from prior SIRT, depicted in figure 1 by following the black arrow. This SBRT plan, labeled SBRT_alone_, is representative of delivering SBRT as a monotherapy, and the limitations that could be involved in treating intermediate and advanced HCC with only external beam therapy. For these plans, PTV volumes were generated by using the original anatomically segmented lesions and applying the same expansion margins as in the SBRT_boost_. To generate an equitable comparison between the SIRT + SBRT and SBRT treatment approaches, SBRT treatment planning parameters and processes were carried over from the SBRT_boost_ planning, including a uniform prescription set to 36 Gy in 3 fractions. Therefore, PTV coverage was favored over sparing of the normal liver volume to meet the planning objectives and de-escalation of the plans was not considered.

### 2.4. SBRT plan comparison

Comparison of the SBRT_alone_ and SBRT_boost_ plans was based on metrics used in our clinic to determine the feasibility and quality of treatment. These metrics include mean healthy liver dose, CV_15Gy_[cc], and RILD NTCP. Additionally, we compared the change in PTV volumes to evaluate the potential target debulking benefit of using SIRT as a pre-therapy to SBRT. For clarification, target debulking is based on SBRT planning volumes created using dosimetric selection of SBRT-eligible lesion volumes compared to SBRT planning volumes created from anatomical lesion volumes.

To calculate NTCP from SBRT, we used a well-established toxicity model, derived from seminal studies on liver tolerance to external radiation performed at our institution (Dawson and Ten Haken 2005, Dawson *et al* 2006). Since reliable NCTP models for SIRT or the combination of SIRT and SBRT have not been demonstrated, only SBRT dose was used for calculations. Given the potentially large amount of NLT irradiated in concurrent treatment of multifocal HCCs, we calculated NTCP using the following parameter values, *n* = 0.97, *m* = 0.12 and a TD_50_ of 35.4 Gy.

## 3. Results

### 3.1. Clinical ^90^Y SIRT study

**Figure 2.**
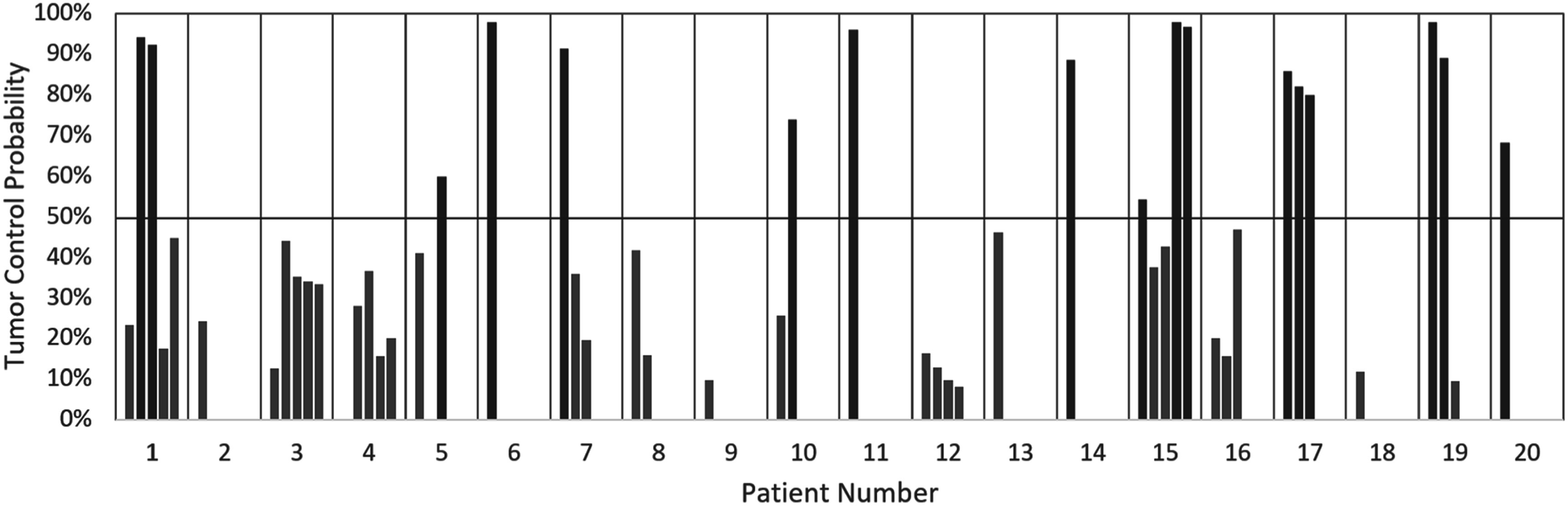
SIRT lesion TCP for all evaluated patients, derived from mean 90Y SIRT dose. The 50% TCP line is highlighted, which was part of the criteria for SBRTboost selection. Multiple bars for a single patient represent multiple HCC lesions. The bars are either highlighted as TCP > 50% (blue) or TCP < 50% (orange), displaying the predicted response of lesions.

Data from 20 SIRT patients with a total of 49 lesions that met the selection criteria were analyzed (data provided in Supplementary Materials, table E1). Across all patients, the mean TD ranged from 5 to 1709 Gy with an average value of 312 Gy ± 290 Gy (mean ± standard deviation). For all lesions, the ^90^Y TCP values as derived from mean TD are presented in figure 2. The average lesion TCP was 47% ± 31%.

### 3.2. SBRT planning study

Considering our criteria of mean TD < 292 Gy (TCP < 50%) from SIRT, 15 of 20 patients had at least one evaluable lesion qualifying for the SBRT planning study. One of these fifteen (patient 13) had a single lesion with TCP < 50%, but no volume within their GTV that received less than 70 Gy, which was the voxel-level threshold determining the area to be boosted by SBRT. This left 14 patients with 31 lesions total eligible for the SBRT_boost_. SBRT_boost_ plans were created for the 14 eligible patients, while the SBRT_alone_ plans for comparison were created for all 20 evaluated patients.

**Figure 3.**
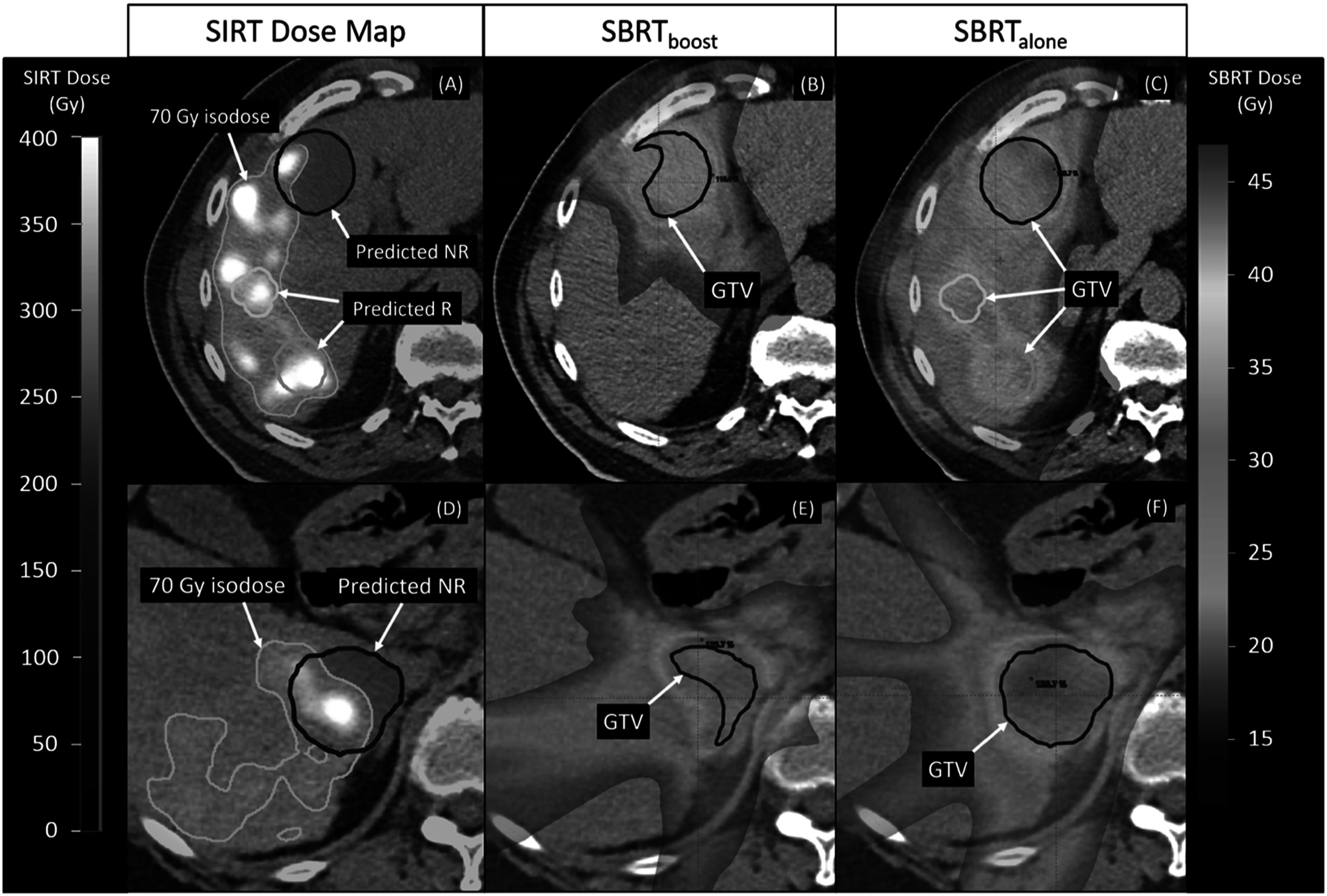
(A) The PET-derived ^90^Y SIRT dose map showing one predicted non-responding (blue) lesion and two predicted responding (green, cyan) lesions following SIRT in Patient 15 (B) For the NR lesion, the SBRTboost only targets a PTV built from all voxels that received <70 Gy. (C) The SBRTalone plan targets all evaluable lesions. (D) The PET-derived ^90^Y SIRT dose map for Patient 2, who had a single predicted non-responding lesion (blue). (E) the SBRTboost plan again targets only voxels with SIRT dose <70 Gy. (F) the SBRTalone plan targets the entire lesion.

Figure 3 shows an example of the PET/CT-derived SIRT dose map and both SBRT plans for patients 2 and 15. The comparison between SBRT_boost_ and SBRT_alone_ dose maps for both patients demonstrate the possible reduction in PTV volume and NLT dose when only targeting predicted nonresponding areas post-SIRT.

**Figure 4.**
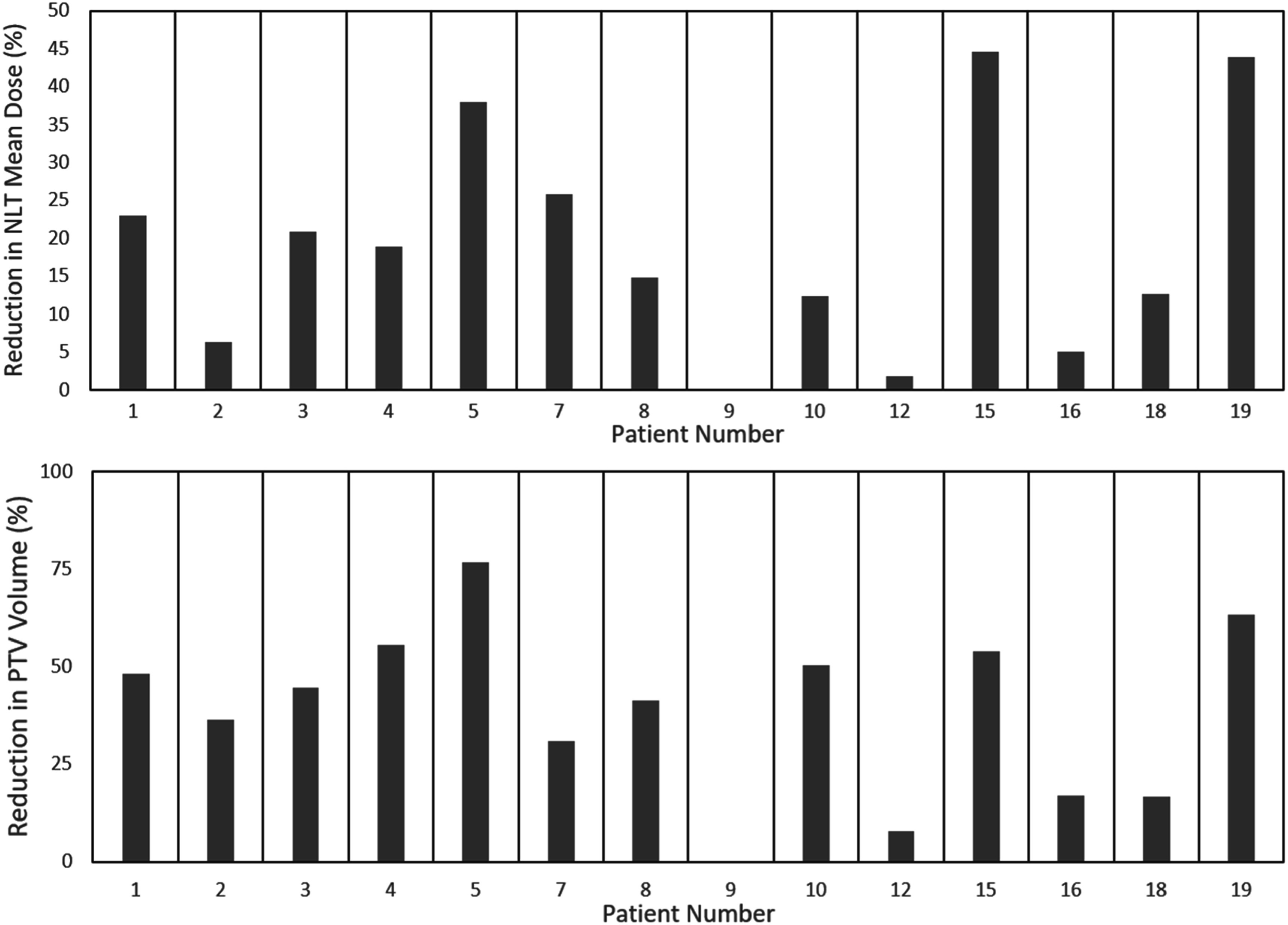
The relative change in NLT mean dose (a) and PTV volume (b) when comparing SBRTboost to SBRTalone. Only the patients that were eligible for SBRTboost plans are displayed. Patient 9 had identical plans since their only lesion had no voxels >70 Gy from SIRT.

The PTV volume and NLT metrics for both SBRT plans across all patients are compared in table 1. Comparing the mean values for SBRT_boost_ versus SBRT_alone_ across the 14 eligible patients, mean dose to the NLT decreased by 2.09 Gy ± 1.99 Gy, CV_15Gy_ increased by 109 cc ± 137 cc, and the PTV volume decreased by 86 cc ± 76 cc. Figure 4 shows the relative decrease in NLT dose and PTV volume between SBRT plans for each boost-eligible patient. Patient 9 shows no difference due to their singular lesion having a maximum SIRT dose <70 Gy, making their SBRT_alone_ and SBRT_boost_ plans the same as they both had to target the entire lesion.

**Table 1.** Comparison of SBRT NLT metrics and PTV volumes between the SBRT_alone_ and SBRT_boost_ plans. Cases where NLT constraints (mean dose < 9 Gy) were not met are bolded/italicized.

	SBRT_alone_				SBRT_boost_				
Patient #	MLD to NLT (Gy)	NTCP (%)	CV_15Gy_ (cc)	PTV Volume (cc)	MLD to NLT (Gy)	NTCP (%)	CV_15Gy_ (cc)	PTV Volume (cc)	Lesions Targeted
1	***10*.4****	0.1	1589	174	8.0	0.0	1860	90	3/5
2	6.0	0.0	1156	69	5.6	0.0	1155	44	1/1
3	***17.6***	97.5	1305	564	***13.9***	9.8	1626	282	5/5
4	***10.6***	0.4	1011	162	8.6	0.0	1124	72	4/4
5	***11.5***	0.6	1643	257	7.1	0.0	2000	59	1/2
6	2.8	0.0	1295	15	N/A	N/A	N/A	N/A	0/1
7	5.5	0.0	1900	42	4.1	0.0	2004	29	2/3
8	8.0	0.0	916	133	6.8	0.0	976	78	2/2
9	4.5	0.0	1016	20	4.5	0.0	1016	20	1/1
10	8.6	0.0	1423	392	7.6	0.0	1456	195	1/2
11	7.1	0.0	878	26	N/A	N/A	N/A	N/A	0/1
12	8.7	0.0	1498	168	8.5	0.0	1484	154	4/4
13	2.1	0.0	1715	41	N/A	N/A	N/A	N/A	0/1
14	6.7	0.0	1405	122	N/A	N/A	N/A	N/A	0/1
15	***17.6***	98.3	750	342	***9.5***	0.0	1216	151	2/5
16	***11.8***	0.5	1116	246	***11.2***	0.1	1163	205	3/3
17	***11.4***	0.2	971	76	N/A	N/A	N/A	N/A	0/3
18	***10.7***	1.8	2028	1195	***9.3***	0.1	2174	997	1/1
19	8.2	0.0	1222	62	4.6	0.0	1418	23	1/3
20	8.1	0.1	1367	250	N/A	N/A	N/A	N/A	0/1

Our clinical standard-of-care is to not exceed a mean dose of 9 Gy to the NLT when prescribing a target dose of 36 Gy in 3 fractions. If this is exceeded, the target dose is decreased, or the number of fractions is increased. 12/20 SBRT_alone_ and 10/14 SBRT_boost_ plans were able to meet this constraint. We were able to meet the goal of CV_15Gy_ > 700 cc in all patients. Patient 15’s boost plans showed the largest absolute reduction in mean NLT dose with a difference of 7.7 Gy, which is due to them having 3/5 lesions that were predicted to respond to SIRT, and thus were not targeted by SBRT_boost_. However, their boost plan was still unable to get below the 9 Gy NLT dose constraint.

## 4. Discussion

To our knowledge, this is the first study to investigate the feasibility of creating SBRT plans based on delivered SIRT dose to HCC tumors. Utilizing ^90^Y PET-derived dose maps and SIRT response models, we retrospectively generated SBRT plans to target regions of tumors underdosed by clinical SIRT, then compared these to a single modality SBRT approach. Feasibility of the combined modalities approach was demonstrated across 20 patients, with 14 patients eligible for SBRT post-SIRT. Of these 14 patients, 10 boost plans were shown to dosimetrically beneficial compared to SBRT_alone_. Furthermore, three of these ten patients had SBRT_alone_ plans that would have initially exceeded our NLT dose constraints, demonstrating ^90^Y SIRT could be used as a first pass tool for downsizing tumors or target debulking in cases where tumor burden or geometry is unfavorable for initial SBRT treatment. Across SBRT_boost_ eligible patients, combination of SIRT and SBRT provided an average reduction in PTV of 39% ± 21% (min: 0%; max: 77%) when compared to SBRT_alone_.

In a retrospective comparison study, SIRT and SBRT have been shown to provide similar overall and disease-specific survival benefit when utilized independently for treatment of unresectable HCC (Oladeru *et al* 2016). Recent SIRT dose escalation studies have demonstrated increased tumor control above certain dose thresholds, indicating that higher SIRT doses are required to improve control in part due to the heterogeneity of ^90^Y microsphere deposition (Chin *et al* 2022). However, dose escalation in SIRT may not be achievable in all cases and could be limited by dose to NLT and the lungs (Garin *et al* 2021). A few studies have demonstrated the safety of performing both SIRT and SBRT in the same patients with relatively long intervals in between treatments (Hsu *et al* 2021, Liu *et al* 2021). Both studies found no major liver toxicities in a total of 22 evaluated patients that received both modalities. As an extension, Abbott *et al* explored creating SBRT plans that consider SIRT dose maps but considered SIRT dose to healthy liver instead of tumor dose. In their planning method, dose was combined from both modalities using a modified BED formula with parameters for SBRT and SIRT that were derived in a previous study. The authors found that SBRT plans could be safely generated while selectively avoiding regions of the NLT that received above a BED-based threshold from SIRT, however they suggested that incorporation of these avoidance regions in SBRT planning resulted in diminished plan quality based on tumor coverage.

In our approach, we explicitly consider disease control from SIRT, reducing target volume size for SBRT planning. This can function to both reduce SBRT dose to NLT while attempting to improve TCP by selectively targeting underdosed regions of the lesions. In our planning approach, we did not consider a toxicity model for combining healthy liver dose between modalities because the appropriate combination of SBRT and SIRT dose and their subsequent combined effects is not-well established and is an area of ongoing research. Additionally, in this study we did not attempt to combine ^90^Y delivered dose with SBRT dose using physical or biological metrics. Recent work by d’Abadie *et al* has demonstrated the potential to combine TCP models from SIRT and EBRT using post-SIRT ^90^Y PET-based tumor equivalent uniform dose, a biological dose quantity rather than absorbed dose (d’Abadie *et al* 2022). However, methods and models that dosimetrically combine SIRT and SBRT still require further development prior to comparing or including them in our proposed treatment strategy due to differences in treatment methods, post-SIRT scanning techniques, dosimetry methods, and reported irregularities in vendor stated activities for the microsphere devices (Abbott *et al* 2020, d’Abadie *et al* 2022, Gnesin *et al* 2022).

In this study, we used a single prescription and fractionation scheme for all patients (36 Gy in 3 fractions) that was based on a standard SBRT treatment protocol for HCC at our institution. For patients who exceed planning constraints under this treatment protocol, clinical care may require dose de-escalation or fractionation changes to reduce normal liver dose or account for other clinical factors (PTV volume and OAR proximity). However, modifications to this prescription and fractionation were avoided in this study to facilitate a standardized comparison between the SBRT_alone_ and SBRT_boost_ plans. For prospective clinical trials, patient geometry and disease extent may warrant modification to this treatment protocol which could impact the liver sparing effect presented in our results. We also assumed a breath-hold or gating technique was possible in all plans, whereas some patients could potentially require additional treatment margins to account for motion in the form of ITV expansions. The choice of PTV or ITV expansion margins would alter the reported decrease in target volumes between the two SBRT plans, but this impact was not evaluated in our study. Furthermore, only VMAT delivery of SBRT was evaluated in this study. Other treatment modalities and delivery techniques, such as protons or MR-guided SBRT with image-based motion gating, may offer increased NLT sparing. Furthermore, due to the retrospective nature of the study, SBRT treatment planning CT scans were not available. Instead SBRT planning was performed directly on the CT from the ^90^Y PET/CT. Therefore, longitudinal changes in patient anatomy between SIRT and SBRT, including those resulting from treatment response and disease progression, were not considered and could impact our SBRT planning results, depending on the time between SIRT and SBRT. Furthermore, these changes along with the need for image registration between the ^90^Y PET/CT and SBRT planning CT could add uncertainty into target creation process. For clinical translation of this treatment strategy, these realities and challenges will need to be considered but are beyond the scope of this in silico trial.

In our proposed sequential treatment approach, we selected a fixed TCP threshold for determining boost eligibility in order to improve control of tumors that were predicted as less likely to respond to SIRT. The selection of specific TCP thresholds contributes to the potential for NLT sparing and improvements in local control, but sensitivity analysis of these thresholds is beyond the scope of this initial feasibility study. To eliminate this sensitivity, we could instead explore an alternative planning approach utilizing the continuous nature of TCP directly in SBRT_boost_ plan optimization. This could result in more uniform combined tumor control and increased NLT sparing by directly accounting for voxel-level SIRT doses and assigning SBRT_boost_ prescription doses at the voxel level through a dose painting approach. However, this would increase planning complexity and may be difficult to implement in currently available commercial treatment planning systems. Furthermore, we do not necessarily expect that our absorbed dose thresholds will be directly generalizable to other centers as it will depend on the usage of glass or resin microsphere, and on post-^90^Y dosimetry methods, including imaging methods and reconstruction processes.

Based on this proof-of-concept planning study, a prospective phase 1 trial has been initiated, where patients are treated with SBRT following SIRT when lesions (or subregions of lesions) are identified as potentially nonresponding using the same dosimetric criteria presented in the current study^4^ 
^4^
Combined Y-90 Selective Internal Radiation Therapy (Y-90 SIRT) and Stereotactic Body Radiation Therapy (SBRT) in Unresectable Hepatocellular Carcinoma (HCC) (https://clinicaltrials.gov/ct2/show/NCT04518748).. To our knowledge, this is the first clinical trial where information from ^90^Y PET/CT imaging-derived delivered dose maps are being utilized in the delivery of SBRT. The combination of SIRT and SBRT has the potential to improve lesion control, extend overall survival, or downstage patients awaiting surgery as compared with either therapy alone.

## 5. Conclusion

We presented a framework for SBRT treatment planning using SIRT dose maps, and demonstrated the feasibility of the approach by retrospectively analyzing patients that received prior SIRT. Hypothetical SBRT boost plans were generated using ^90^Y PET/CT-derived dose maps and SIRT response models to guide selection of treatment volumes, and were compared to hypothetical SBRT plans assuming no combination of therapies (ignoring prior SIRT). We demonstrated and quantified the degree to which SIRT dosimetry guided SBRT plans lead to a decrease in PTV volumes, normal liver dose, and NTCP compared with SBRT plans that did not consider SIRT. Demonstration of the safety of this approach is the focus of an ongoing prospective clinical trial.
